# How can we overcome health inequalities in psychiatry?

**DOI:** 10.1192/bjb.2023.49

**Published:** 2023-08

**Authors:** Jonathan Monk-Cunliffe

**Affiliations:** Centre for Academic Mental Health, University of Bristol, Bristol, UK

**Keywords:** Clinical governance, economics, epidemiology, psychosocial interventions, social deprivation

## Abstract

Health inequalities in psychiatry are well established, with people living in poverty and those from minoritised groups receiving different care and experiencing worse health outcomes. Psychiatric patients experience significant differences in life expectancy compared with the general population. This article explores changes within psychiatric services and public health interventions that could address health inequalities and asks why this has not happened yet.

We know that poverty and marginalisation cause mental illness and affect some groups more than others. We know that people from minoritised groups, be that based on ethnicity, sexuality or gender identity, receive different care and have different health outcomes.^1^ We also know that people with severe mental illness die, on average, 20 years younger than the general population.^2^

There is a wealth of evidence for interventions at a service and population level that can address these inequalities, and the moral, ethical and economic reasons for doing so are well established. Yet, psychiatry has failed to effectively overcome these stark facts. So – what needs to change?

## Within psychiatry

As psychiatrists we hear the stories behind the statistics of how poverty and marginalisation contribute to and perpetuate mental illness. It's our job to understand how complex factors combine and to think about how to support someone, but in doing so we put too much responsibility on individuals to make up for system failures. We ask patients to get better, while they remain in the social conditions that made them sick, and we ask psychiatrists to treat people, while working in services stretched to the limit.

The elephant in the room is money. In England this year 13.8% of local National Health Service (NHS) spending is allocated to mental health (including intellectual disabilities and dementia),^3^ whereas mental disorders contribute to 16% of the disease ‘burden’, based on international data from 2019,^4^ before any increase in demand following the COVID-19 pandemic has been factored in. Without appropriate funding to match need we will not overcome health inequalities in psychiatry. Appropriate funding translates to sufficient staffing and case-loads that give us the time to think with our patients about how we can support them in a way that addresses their specific needs.

It also translates to having time to reflect on how we structure our services, which is not possible when clinical leaders are trying to stop the ship from sinking. With time to think we can first identify and then address inequalities in access to healthcare, patient experience and health and social outcomes. The Advancing Mental Health Equality (AMHE) framework sets out ways that local commissioners can develop plans to tackle mental health inequalities in their area, with practical suggestions for how to implement change and support marginalised groups, for example through outreach services or culturally adapted versions of evidenced-based treatments.^1^ We know that minoritised ethnic groups, LGBTQ+ people and homeless people have a different experience of healthcare, and that layers of oppression can be compounded through intersectionality. Listening to patients and carers and co-producing services could develop routes to care that are more acceptable and accessible. The first contact with mental health services for too many people from Black and Asian communities is being detained under the Mental Health Act.^5^ Reforms to the Act could start to address this, but are long overdue, in particular reviewing community treatment orders, used 11 times more often for people from Black and ethnic minority groups.^6^

Physical health is another area where inequality is striking, with the shameful life expectancy gap of 20 years for people with severe mental illness.^2^ We must think about how to support our patients with evidence-based interventions for obesity, smoking and substance use, and ensure that we collaborate with general practitioners and other colleagues to monitor the side-effects of medications we know can cause iatrogenic harm.

The old adage that prevention is better than cure remains elusive in practice, but is essential if we want to address health inequalities. The Public Mental Health Implementation Centre (PMHIC) has collated the evidence for population-level interventions that could improve mental health.^7^ They are wide-ranging, including family centres to support with pregnancy and parenting, childhood education programmes promoting social and emotional learning, smoking bans and alcohol pricing, and workplace interventions to reduce stress. However, evidence for what helps some of the most vulnerable people is limited, as groups are excluded from research as well as from services.

## Beyond psychiatry

The things that the PMHIC suggests are not unique to psychiatry. Michael Marmot has consistently identified support in early years, education, employment and a healthy standard of living as being essential for tackling health inequalities and improving health.^8^ Population interventions to reduce mental illness would not just benefit our psychiatric patients, they would create a happier, healthier society across the board. Preventing illness and facilitating healthier lifestyles will also help tackle the climate crisis, which has mental health impacts that affect deprived and marginalised communities more than others and will exacerbate existing socioeconomic inequalities.^9^ However, despite knowledge of what to do and why, we see that things don't change. To address this we must move beyond the remit of psychiatry and to an even more complicated P: politics.

Many of the changes suggested fall outside the budget of health and into other areas. Government departments work in silos, with education judged by how it improves exam results, not by how it improves health. Policy is short-sighted, limited by the terrifying acceleration of electoral cycles. It's much easier to take a photo next to a new hospital to show voters you've helped them than it is to address social determinants and have someone else reap the rewards at the ballot box 20 years down the line.

As individuals we can't do this alone. We need a strong College to make our collective voice heard and advocate for psychiatry and our patients in an effective manner. This must include appropriate funding for mental health services, to give clinicians time to deliver the care our patients deserve and to have the space to reform our services to address inequalities in access and experience. It is encouraging to see leaders highlight the social causes of mental illness,^10^ but we must call for action beyond healthcare reform and join with other health professionals to persuade the government to adopt policies that will stop trapping people in conditions that make them sick and enable them to exercise their human right to health.

There is only so much that we can do as psychiatry, and what we can do ourselves won't be enough to fix the problem. The simple answer to this essay's question of how can we overcome health inequalities in psychiatry is that *we* can't. As psychiatrists we have a responsibility to support and advocate for our patients, but there are limits to our role, and if we want to see health inequalities abolished we must each decide as citizens whether we want to join a broader coalition of voices shouting for change.

We are well placed to step up to this task, should we choose to, with an understanding of how to distil and communicate complex problems. We can use our platforms to amplify the voices of patients and those at the sharp end of inequality, who know most about how things must change. Organisations, such as the charity Medact, offer the opportunity to connect with others in the health community campaigning for a better, fairer world and can help to stem some of the angst that comes from climbing the mountain alone.

## Conclusions

We've known for a long time the pernicious effect of health inequalities in psychiatry and we know what needs to change for them to be overcome. Appropriate funding would give time and space to reform psychiatric services and refine our practice, while advocating for population level interventions to reduce inequality. This will get us so far, but as psychiatrists we cannot address inequality alone. If we want to do that we must each decide to join a broader coalition standing for change.

## Data Availability

Data availability is not applicable to this article as no new data were created or analysed in this study.

## About the author

**Jonathan Monk-Cunliffe** is a National Institute for Health and Care Research (NIHR) Academic Clinical Fellow and Year 3 Specialty Trainee (ST3) in Psychiatry in the Centre for Academic Mental Health at the University of Bristol, UK.
